# Human Computer Interactions in Next-Generation of Aircraft Smart Navigation Management Systems: Task Analysis and Architecture under an Agent-Oriented Methodological Approach

**DOI:** 10.3390/s150305228

**Published:** 2015-03-04

**Authors:** José M. Canino-Rodríguez, Jesús García-Herrero, Juan Besada-Portas, Antonio G. Ravelo-García, Carlos Travieso-González, Jesús B. Alonso-Hernández

**Affiliations:** 1 Signals and Communications Department, University of Las Palmas de Gran Canaria, Las Palmas de Gran Canaria 35001, Spain; E-Mails: antonio.ravelo@ulpgc.es (A.G.R.-G.); carlos.travieso@ulpgc.es (C.T.-G.); jesus.alonso@ulpgc.es (J.B.A.-H.); 2 Computer Science Department, University of Carlos III, Madrid 28903, Spain; E-Mail: jesus.garcia@uc3m.es; 3 Signal, Systems and Radio-communications Department, Polytechnic University of Madrid, Madrid 28040, Spain; E-Mail: besada@grpss.ssr.upm.es

**Keywords:** aircraft navigation procedures, navigation management system, HCI, air traffic system, smart environment

## Abstract

The limited efficiency of current air traffic systems will require a next-generation of Smart Air Traffic System (SATS) that relies on current technological advances. This challenge means a transition toward a new navigation and air-traffic procedures paradigm, where pilots and air traffic controllers perform and coordinate their activities according to new roles and technological supports. The design of new Human-Computer Interactions (HCI) for performing these activities is a key element of SATS. However efforts for developing such tools need to be inspired on a parallel characterization of hypothetical air traffic scenarios compatible with current ones. This paper is focused on airborne HCI into SATS where cockpit inputs came from aircraft navigation systems, surrounding traffic situation, controllers’ indications, *etc.* So the HCI is intended to enhance situation awareness and decision-making through pilot cockpit. This work approach considers SATS as a system distributed on a large-scale with uncertainty in a dynamic environment. Therefore, a multi-agent systems based approach is well suited for modeling such an environment. We demonstrate that current methodologies for designing multi-agent systems are a useful tool to characterize HCI. We specifically illustrate how the selected methodological approach provides enough guidelines to obtain a cockpit HCI design that complies with future SATS specifications.

## 1. Introduction

The current air traffic system is a centralized Air Traffic Control (ATC) where air traffic controllers normally use voice communication links to give aircraft clearances (e.g., heading, altitude, speed, *etc.*) to maintain a safe distance between them. The controller decision-making process is based on tactical decisions to separate aircraft taking into account information from their radar positions and filed flight plans (planned flight route information) [1]. This air traffic control scheme is not efficient enough to support a substantial increase in air traffic as is forecast for the next 20–25 years [2,3].

On the other hand, nowadays a new technology support for Communications, Navigation, Surveillance and Air Traffic Management (CNS/ATM) are being developed for civil aviation [4]. The CNS/ATM system will allow the sharing of real-time data between different agents involved in air traffic (aircraft, air traffic separation control and other air traffic service providers, airlines, *etc.*). Thus, aircraft can share their on-board sensors and navigation data (position, speed, course, intended flight plan, predicted trajectory, airborne weather conditions, *etc.*) with or through other agents. Aircraft can also have access to real-time information provided by several air traffic services (weather, navigation resources, air-traffic conditions, *etc.*). Then, the ability to share information, along with the more flight routes based on satellite navigation, suggests the need to develop new methods and systems for both air navigation and air traffic management in order to achieve their aims in a more efficient way [5]. 

Several research initiatives, that are currently underway, are aimed at implementing a more strategic air traffic control based on predicted four-dimensional (position plus time) aircraft trajectories (4D Trajectory Base Operations or TBO concept [6,7]). Thus, SESAR (Single European Sky for ATM Research) and Next-Gen (Next-Generation Air Transportation System) projects are the two prior initiatives that manage several research projects intended to develop technological infrastructure for the next generation of Air Traffic Management (ATM) systems under a TBO vision [7,8,9]. 

However, effective implementation of this new operational concept still requires advances on several pending issues related to:

(a) New coordination procedures for distributed decision-making processes in order to make aircraft preferred trajectories compatible with a well-organized air traffic [10,11,12,13]. It requires the definition of new roles of the Smart Air Traffic System (SATS) agents and specific communication protocols for automated processes such as: (i) air-ground and air-air trajectories negotiation; (ii) managing shared information from aircraft and air traffic service providers; (iii) monitoring aircraft states and intentions from airborne (or navigation) and from ground (or air traffic control) perspectives; and (iv) solving unexpected events during the procedure execution.
(b) New HCI designs that allow SATS users (mainly aircrew, air traffic controllers) to carry out their respective tasks for different automation levels [14,15] of above procedures. In addition, these HCI require using top-level languages to achieve a precise intercommunication of trajectory-related information between aircraft systems and ground systems. These languages should enable human-readable comprehension of inter-machine communication processes [16].
(c) New underlying mathematical models and algorithms required by the mentioned air and ground systems. These represent the computational side of HCI and they must implement several functions related to the management of trajectory and parallel decision-making processes (e.g., trajectory synthesis and prediction [17,18,19], trajectory conflict detection and resolution [20,21], on-board four-dimensional trajectory guidance [22], *etc.*). Data used by these models provide mainly from sensors inputs, intercommunication systems and human-machine interfaces (HMI) [23,24].

According to current frameworks for HCI design [25,26], the obvious high interdependence between the previously mentioned open issues makes it extremely difficult to outline a preliminary independent design of procedures, HCI systems and their underlying computation models.

In this context, our work is focused on analyzing airborne activities for designing HCI for SATS aircraft navigation systems. The work approach takes into account the above interdependences in order to include them into a SATS conceptual model that provides enough specifications for designing such HCI systems.

### 1.1. Previous Works

Several works have been proposed to provide a solution for the design operational procedures, human-machine interfaces (HMI) as well as underlying computational supports.

Regarding the design of procedures, the Distributed Air-Ground Traffic Management (DAG-TM) [10,11,27] project provided a framework based on several air traffic scenarios from where roles of aircrew and air traffic service providers were defined. However no specific operational procedures have being subsequently developed. 

Besides, numerous experiments proved feasibility and beneficial to different HMI and support tools for decision-making for precision space separation in such environments [28,29,30]. Some examples of these tools for aircraft are: the Airborne Separation Assurance System (ASAS) [31], Cockpit Display Traffic Information (CDTI) [32,33] and Flight Management Systems (FMS) with four-dimensional (4D) trajectory guidance capabilities [34,35] (current FMS are able to track flight routes defined by tri-dimensional way-points -3D FMS- or way-points with Required Time of Arrival -3.5D FMS-). Moreover, some preliminary proposals for automated air-trajectory synchronization and negotiation have been evaluated [36,37]. Parallel to that, mathematical models and tools for trajectory predictions [18,38] and detections and resolution the conflicts where studied (e.g., [19,20,39,40]). Nevertheless new high-level functionalities for automatic coordination between aircraft systems and between these ones and other air traffic service providers are required. Other approaches have focused on simulation of air traffic scenarios with different levels of fidelity to develop and evaluate new operational concepts (e.g., [41,42,43,44]). Experimental results of these works indicate that advanced ATM concepts make a sound case for next generation air-traffic systems; however, there is a need to investigate and understand their complex interaction under non-nominal scenarios [42]. 

Moreover, the dynamic nature of air traffic and its geographical and functional distribution have attracted the attention of agent researchers since the last decade to study new inter-agent coordination schemes for automated air traffic scenarios. Thus the Multi-Agent Systems (MAS) theory has been used as a suitable framework to analyze and model the organization of autonomous air traffic entities that coordinate and negotiate their actions in order to achieve their particular goals [45,46,47,48,49,50]. However, results of attempts for developing these simulation and analysis tools to address the paradigm shift in navigation and air traffic procedures show the need for more detailed and structured conceptual models to support these tools. These conceptual models should provide a comprehensive description of cited interdependences.

More recently SESAR projects outlined a timescale for defining, developing, validating and deploying several Air Traffic Management capability levels [9]. Consequently SESAR integrates and coordinates results of previous efforts for developing new operational procedures and associated systems. Therefore, it is an extensive long-term coordination plan for the research community. It suggests that designing HCI could be significantly expedited by means of a methodological approach for analyzing user tasks in hypothetical future air traffic scenarios. 

The above works approaches can be classified according to the viewpoint used for analyzing and modeling the SATS and its elements. Two main perspectives can be identified: the abstraction level and the dynamic *vs.* static perspective. Three abstraction levels have been taken into account in referred literature: (i) a high or macroscopic level that describes different air-traffic scenarios and required functionalities; (ii) an intermediate or mesoscopic level that describes roles of SATS agents and their interactions; and (iii) a low or microscopic level that describes internal processes of each particular agent and its detailed architecture.

On the other hand, dynamic behavior of the SATS is defined by the interdependence between air-traffic scenarios, agent interaction protocols and their inner processes. The static or structural view defines functionality specifications, SATS overall architecture and agent structure.

Therefore we propose to develop a multidimensional conceptual model that integrates mentioned perspectives. The development of this model requires a methodological approach to provide consistence and systematic cross-check between model components. As a result of this approach, procedures and the corresponding air and ground systems functionalities for SATS should be modeled. Moreover, the model obtained could be used as support for developing simulation tools intended for evaluating and validating such procedures.

### 1.2. Our Working Approach

In this work we use recent proposals in agent-oriented methodologies as a suitable approach for analyzing new air traffic scenarios. These scenarios are regarded as a result of the dynamic behavior of SATS. Then, a SATS model should define: roles and rules for agent-interactions (procedures), an inner agent architecture (HCI architecture) and mathematical models and algorithms for performing agent computation. We specifically focus our work on applying this approach for modeling a functional architecture in a HCI system for next-generation cockpit where inputs coming from sensors and other sources are conveniently modeled and represented to take part in the design [23,50]. 

This approach is also consistent with other approaches that propose making use of scenario-based on human-computer interactions [51] and other HCI methodologies based on the activity theory [25,26].

Fortunately, the current state of the art of multi-agent technologies provides methodological approaches and tools to develop such models. It is now possible to apply practical and formal methodological approaches in state-of-the-art agent technology to analyze the following issues, in a structured and consistent manner: roles and functionalities of autonomous entities (agents) that take part in an operational scenario; interactions between entities (agents’ coordination and communication protocols) and, inner architecture and dynamic behavior (processes) of entities. A systematic integrated design may derive from this analysis. Several multi-agent methodology approaches have been proposed in recent years and some comparative analysis are beginning to appear in literature [52,53,54,55]. *Prometheus* is a well-established agent-oriented methodology, which we selected to provide guidelines to develop a SATS as a multi-agent system [56,57].

*Prometheus* is well suited for solving our problem due to:

(a) highly detailed guidelines for the initial system specifications;
(b) the modularity of the agents’ internal architecture supported by the concept of capabilities (so creating a direct relationship between the description of these capabilities and the functionalities/functions of different aircraft and ground systems); and
(c) the option of translating conceptual model specifications into working model by means of representative agent-development platforms such as JADE [58], JADEX [59], JACK [60], *etc.*

### 1.3. Paper Organization

This paper is organized as follows: Section 2 illustrates how relevant concepts of the Prometheus methodology can be adapted to analyze and design a conceptual SATS model. Then the detailed design of an aircraft agent results in a capabilities-based cockpit architecture (Section 3). Capabilities are formally defined within the Prometheus framework as agent modules used to separate processes into individual components. These cockpit capabilities extend current functionalities of Flight Management Systems (FMS) and Autopilot/Autothrottle (AP/AT) [61] systems to achieve a higher automation level for future cockpit navigation systems. Later, as a result of previous analysis and design, a cockpit system architecture is described in Section 4. Finally, conclusions are presented in Section 5.

## 2. Applying Agent Methodology for Modeling Smart Air Traffic Scenarios

**Figure 1:**
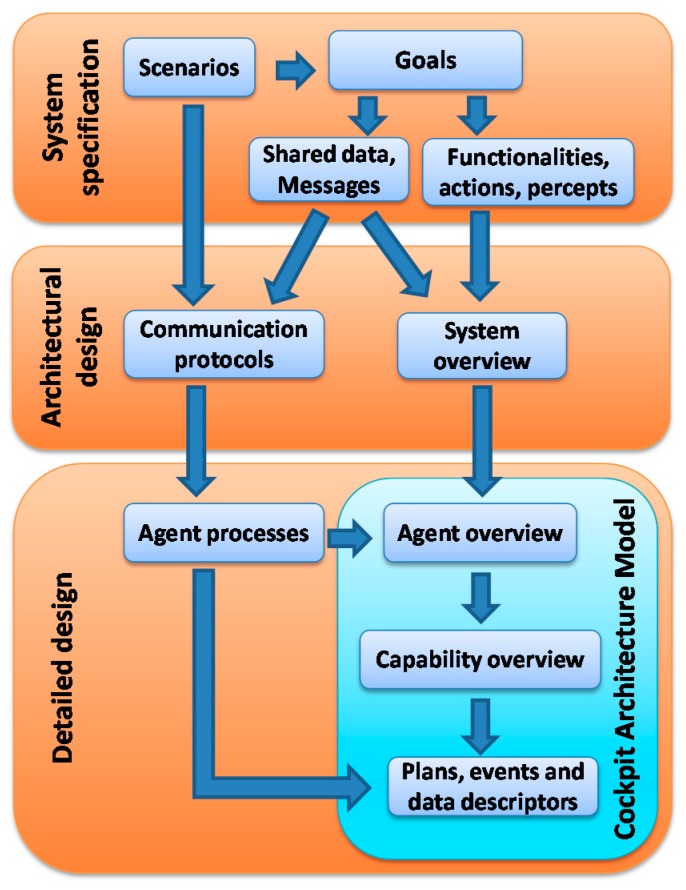
Methodological Approach.

Following the proposed methodology, our conceptual model has been developed through an iterative process carried out in three phases (see Figure 1): system specifications, architecture design and detailed design. 

The SATS specifications and architectural design phases as a multi-agent system provide enough information to achieve a detailed design of its corresponding agents: *i.e.*, aircrafts, air traffic controls, *etc.* Later on, this information is used in this work to complete a more detailed design centered on the aircraft agent. Within this agent model, underlying mathematical and algorithms required for performing specific tasks are defined as agent’s plans. Also, plans with similar goals are grouped into capabilities that model specific cockpit HCI components. 

The structured nature of elements at different abstraction levels identified in each one of these phases facilitates crosschecking for completeness and consistency of the model as will be seen below.

### 2.1. System Specification

The system specification starts by identifying several uses cases (or scenarios) that take part in the overall air traffic scenario. These scenarios illustrate essential aspects of system operations and they can be split into new sub-scenarios at different levels. In order to identify the most representative scenarios, it is useful to associate goals to each of them. Then, scenarios and goals help analyze the main system’s functionalities (*i.e.*, abilities that the system needs to achieve some of its goals) and the system-environment interface defined in terms of system inputs (percepts) and outputs (actions)*.*

In a SATS percepts are inputs from the human side of the HCI or data from environmental sensors. So airborne system inputs (aircraft agent percepts) must integrate both the on-board navigation sensors and the external inputs from ATC and other surrounding aircraft so that the pilot has all the important information for taking decisions.

In the same way, actions represent outputs to the human side of the HCI (e.g., visualization data) or other dynamic behavior of the SATS agents (e.g., aircraft movement)

The base scenario proposed to obtain the conceptual model of a SATS is a distributed process where several autonomous and proactive entities (agents) plan and execute a set of coordinated tasks to provide free-of-conflict 4D trajectories. Moreover, we focused this scenario on the arrival and approach flight phase because this one presents particular restrictive and complex flight conditions as well as greater coordination requirements. Initial guidelines for defining an arrival and approach air traffic scenario are based on the Distributed Air-Ground Traffic Management (DAG-TM) project [27]. According to the referred guidelines, the flight crew: (i) could negotiate arrival-preferred trajectories with ATC; and (ii) is responsible for maintaining longitudinal spacing between consecutive aircraft once a trajectory (or trajectory constraints) has been assigned. 

From this operational scenario, the following types of agents can be identified: Aircraft, Air Traffic Control (ATC), Airline Operational Control (AOC), Meteorological Service Provider (MPS) and Airspace Resources Provider (ASP) [27]. Then, first level scenarios are constituted for scenarios that take into account each agent’s perspective. Thus it is possible to define the following sub-scenarios: Manage Aircraft, Manage ATC, Manage Airline Operational Control, Provide Weather Information and Provide Airspace Resources. 

**Figure 2:**
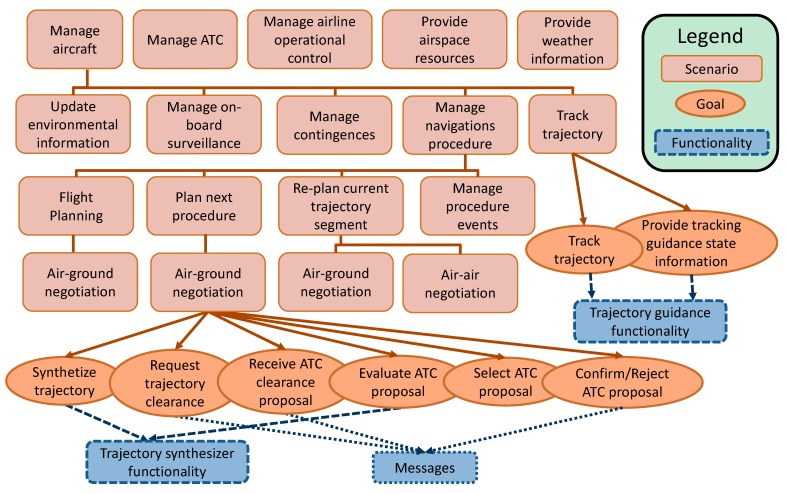
System Specification from aircraft scenarios: goals and functionalities identified from “trajectory guidance” and “air-ground negotiation” sub-scenarios.

Consecutively, each of the previous scenarios can be broken down into several sub-scenarios. Besides, some of these sub-scenarios can be part of different root scenarios (see Figure 2). Hence focusing on the *Manage Aircraft* scenario and taking into account on-board processes, it can be split into the following: *Update Environmental Information*, *Manage On-Board Surveillance, Manage Contingencies*, *Manage Navigation Procedures* and *Track Trajectory.*

From this analysis, the *Manage Navigation Procedures* sub-scenario is, in the proposed approach, a key scenario for defining new on-board HCI functionalities. However, the rest also needs to be taken into account due to their relationship with this one as will be explained later. 

For a more detailed expansion of *Manage Navigation Procedures* scenario, several on-board tasks related to planning, implementation or modification of trajectory for different flight phases need to be analyzed. 

In this context, a trajectory can be expressed here as a sequence of 4D points space-time constraints (4D trajectory), vector instructions (e.g., heading, speed and/or altitude until the next point), aircraft intent (e.g., a route with specific arrival times restrictions) or a combination of them. 

We also define a navigation procedure as a set of airborne tasks aimed at flying a determined flight phase. Therefore, a gate-to-gate airborne trajectory based operation consists of a set of main flight procedures (taxi, departure, in-route, arrival/approach, landing, *etc.*) that must be planned and executed (and sometimes modified) in a sequential way (procedures list). Some alternative procedures can also be defined for each flight phase in order to manage abnormal and emergency situations. 

In addition, procedures must be characterized by specific attributes or properties such as: state, conditions and constraints for performing tasks (e.g., spatial and deadline restrictions), trajectory data, *etc.*

The procedure states can be also classified in the following manner:

- *Planning*_,_ when procedural tasks are intended to calculate and negotiate the trajectory.
- *Executing*, when procedural tasks manage parameters of a running trajectory.
- *Re-planning*: tasks that are aimed at performing a trajectory modification to resolve contingences that arise during a procedure.

**Figure 3:**
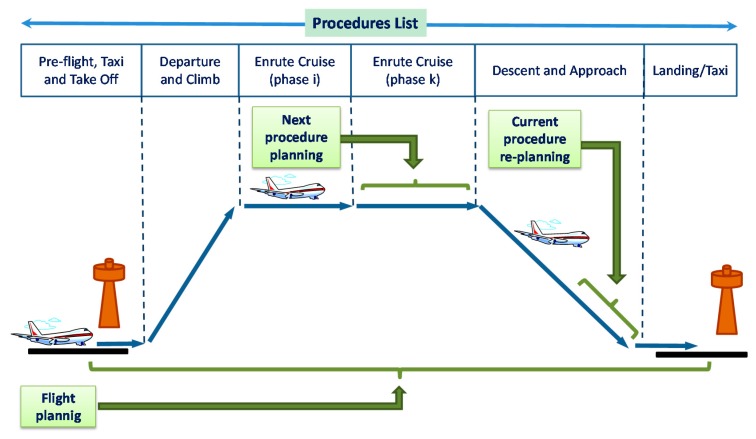
Gate-to-Gate Flight Procedures List.

Then, in a sequence of gate-to-gate procedures (see Figure 3) the on-board planning activities in each phase are for planning the overall flight, planning the next flight phase (for updating trajectory data) or re-planning the current flight phase (when contingences arise).

Taking the above considerations into account, activities of the *Manage Navigation Procedures* scenario can be analyzed from the next four sub-scenarios:

(a) *Plan Flight-Plan* scenario including calculation and communication processes in order to plan the flight trajectory for the overall flight (*i.e.*, obtaining initial data for trajectory and time-space constraints to negotiate updated trajectories for each flight phase).
(b) *Plan Next Procedure* scenario that performs/implements the trajectory planning process to update the trajectory and other attributes for the next flight phase.
(c) The *Re-Plan Current Procedure* scenario that performs partial modifications for the current trajectory in current flight phase when airborne contingences arise.
(d) *Manage Procedure Events* scenario that analyzes the current procedure and generates events to implement trajectories (or partial trajectory modifications) and initiate the next procedural planning.

Planning activities will include the coordination process in order to obtain information from other agents or for negotiating a specific trajectory with them. Therefore, these activities are included in new sub-scenarios that are also shared with these other agents. 

In order to simplify this description in Figure 3, the scenarios structure described above is summarized. In this scheme, we focused on the negotiation processes because they are the best representation of next-generation airborne capabilities for automated coordination. As a result, *Plan Next Procedure* scenario contains *Air-Ground Trajectory Negotiation* scenarios. In the same way, the *Re-Plan Current Procedure* scenario contains sub-scenarios to illustrate air-air negotiation and air-ground negotiation required to modify a trajectory under execution. 

For above scenarios architecture, several functionalities can finally be identified. As was explained, a practical way to recognize these functionalities consists of associating specific goals to each scenario. Then, as it is shown in Figure 3, main aircraft functionalities related to a trajectory can be recognized from lowest level scenarios goals. Some of these functionalities are as follows: trajectory prediction, trajectory conflict detection, trajectory performance evaluation, trajectory tracking, *etc.* In addition, other functionalities are also required for managing inter-agent communication messages.

At this design stage, actions and percepts can also be defined. For the aircraft agent, actions consist of the aircraft movement and outputs for graphical pilot interfaces. Percepts come from aircraft sensors and from the pilot-input interface (options menus, flight and power controls, *etc.*).

### 2.2. Architecture Design

As result of the previous specification system phase, this architecture design phase captures the static organization of the overall SATS as well as its dynamic behavior.

- The overall system structure (static) can be depicted in a system overview diagram that links agents, showing interaction protocol names, data used by each agent as well as agent percepts and actions.
- The dynamic behavior system can be represented by a detailed design of interaction protocols or individual communication routes between agents. Protocols capture the timing of message communications between agents [56,62,63].

**Figure 4:**
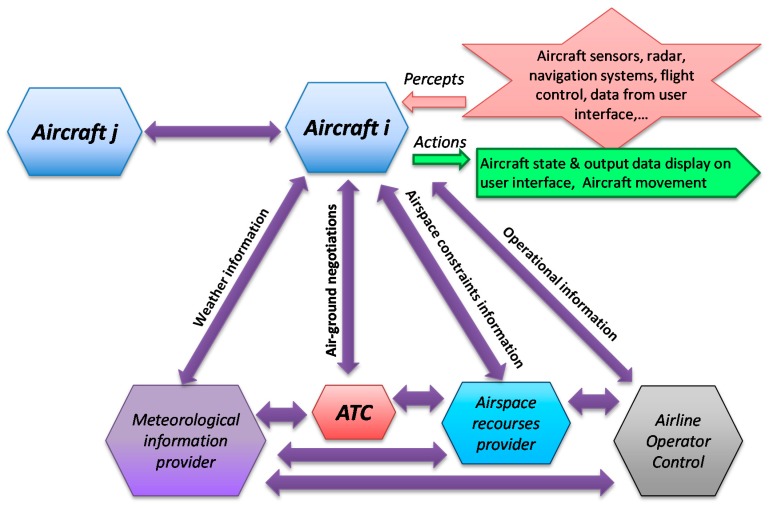
Simplified System Overview.

Figure 4 shows a simplified representation of the system overview diagram, focused on the aircraft agent. Main interactions of this agent with the environment (percepts and actions) and with other agents (communication protocols) are shown. Communication protocols allow the aircraft to improve its knowledge base about the environment and other agents’ intentions as well as negotiating trajectories that could be in conflict. 

**Figure 5:**
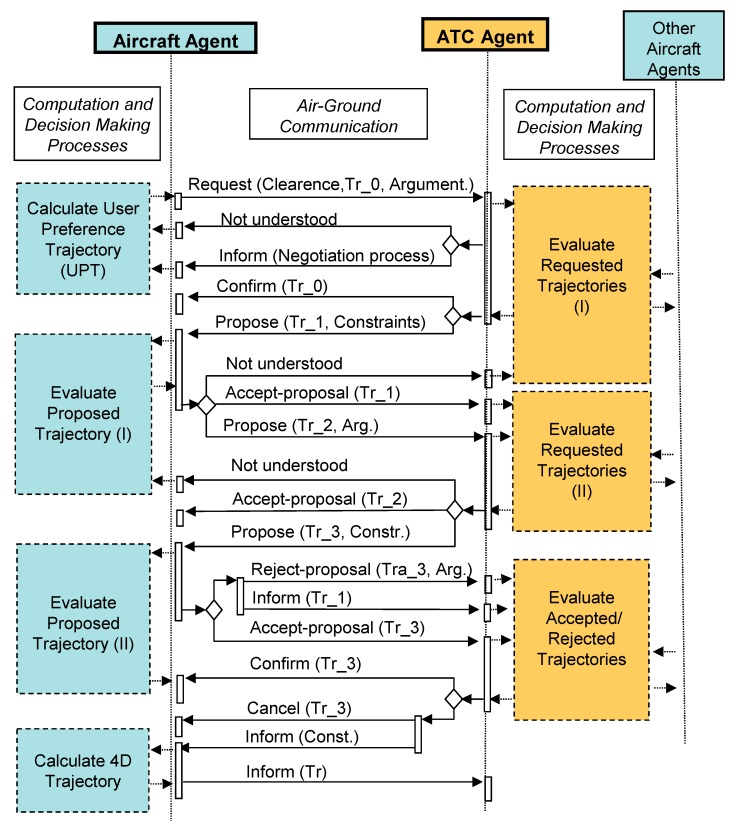
Air-Ground Negotiation Protocol Example.

In order to illustrate aircraft agent inner processes when a planning procedure is being implemented, Figure 5 shows an example of basic air-ground negotiation protocol to negotiate arrival trajectories. Protocols like this one represent the core of a planning process for an arrival/approach procedure. This planning process for arrival and approach procedure (and therefore the mentioned protocol) is activated by a specific event occurring during the execution of previous en-route navigation procedures. Although a new negotiation scheme can be defined from this design, all of them will use similar functionalities to evaluate proposals and generate counter-proposals during their respective decision making-processes. Therefore, this protocol and its associated functionalities provide guidelines and enough specifications for developing new aircraft and ATC coordination procedures.

In Figure 5, on board computation processes while aircraft negotiate their preferred trajectories are represented on the left side of the aircraft agent lifeline. Furthermore, the computations performed by ground systems are depicted on the right side of the ATC agent lifeline. In the center of the figure, inter-communication messages are presented. These messages could be performed by a normalized FIPA support [64].

Then through these protocols aircraft can:

- Request clearance to perform its preferred trajectory and arguments.
- Accept or reject ATC trajectory proposals.
- Perform counter-proposals to ATC agent.
- Inform about content of final decision adopted.

At the same time, ATC can:

- Confirm trajectories requested by aircraft.
- Propose alternative requested trajectories.
- Accept or reject aircraft proposals.
- Inform about specific content of the final decision adopted.

### 2.3. Detailed Design

In the detailed design phase, the internal agent processes and the internal agent architecture are built taking into account previous sections of design phases. A process represents the agents’ local view for an interaction protocol. 

At computation level, functionalities and other agent processes can be implemented by mean of *plans*. A *plan* is a sequence of simple tasks that represent a specific way of responding to an event. Events consist of the arrival of a percept, arrival of a message from another agent or an internal message in the agent. Plans to implement specific functionalities and decision-making and inter-agent coordination processes are grouped into capabilities. Therefore, in this case, capabilities represent the computational side of the HCI system and are considered as restructuring mechanisms akin to modules that implement several interrelated functionalities and processes by means of plans. Some capabilities can also be broken down into sub-capabilities before being defined at the lowest level in terms of plans, events and data. 

**Figure 6:**
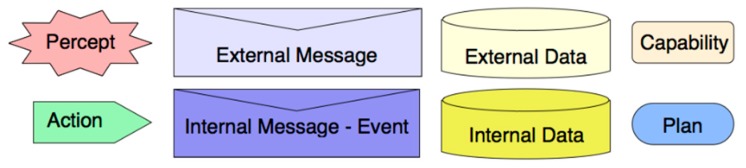
Notes used in agent and capability overview diagrams.

Thus, the agent architecture is defined by several capabilities that exchange data by means of inner or external messages. The notes used to represent previous components into the agent architecture are depicted in Figure 6 and the aircraft agent design is summarized in the next section.

In the next section, these methodological guidelines are applied for designing the aircraft agent.

## 3. Aircraft Agent Design: Main On-Board Capabilities

The functionalities identified in the specification system phase can be grouped into the following six cockpit capabilities:

(a) *Aircraft Environment Information Management.*
(b) *Aircraft Systems Alarm Management.*
(c) *Conflict Detection-Resolution.*
(d) *Airborne Contingency Management.*
(e) *Trajectory Guidance.*
(f) *Navigation Procedures Management.*

Most of the previous capabilities contain functionalities that derive from the scenario structure proposed in the specification phase. 

Taking into account that next-generation of airborne navigation systems will be based on an on-board automated procedure management, the *Navigation Procedures Management* capability is the core of the cockpit HCI. The rest of the capabilities provide environmental and airborne data and generate events for this capability. 

**Figure 7:**
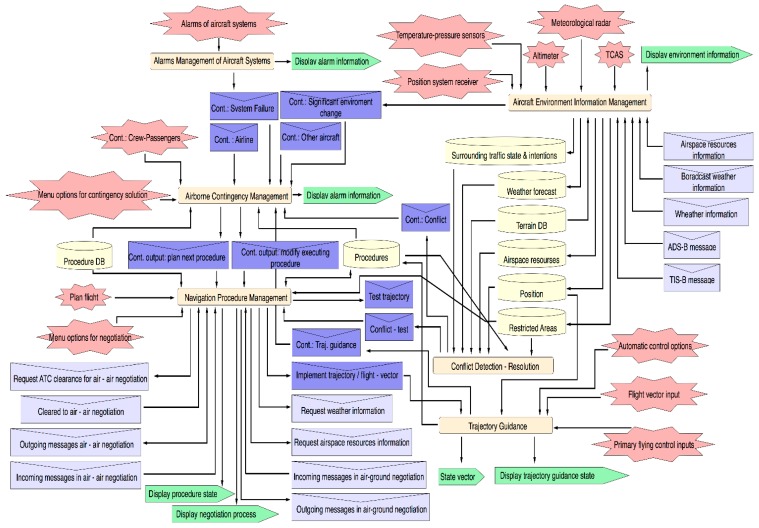
Aircraft Agent Architecture.

Above capabilities are represented in the Aircraft Agent Architecture in Figure 7. In that figure, basic components of this agent are depicted (e.g., percepts, actions, shared data, inner inter-capability messages and external inter-agent messages). Inner messages usually contain information about specific contingences that arise in flight. External messages are part of the agent’s coordination processes.

In the following subsections, a summarized description is presented.

### 3.1. Navigation Procedures Management

This capability contains plans for managing trajectory planning processes. In Figure 6, main perceptions, actions, data and messages associated to these capabilities are indicated.

Percepts (like Flight Plan or Menu Options for Negotiation) allow the crew to take part in making decisions, by interior messages from other capacities that contain data for managing planes. Actions display several types of data about the negotiation proposals or task status for planning and executing specific trajectory segments. 

Plans that implement referred tasks are organized in four sub-capabilities (*Planning Flight, Executing Procedure Events, Planning Next Procedure* and *Re-Planning* *Current Procedure)* related to the four sub-scenarios for the *Manage Navigation* scenario described in Section 2. 

*Flight Planning* contains plans for preparing and coordinating tasks aimed at determining an initial gate-to-gate trajectory.

*Executing Procedure Events* capability generates events and data for starting plans for new trajectory planning processes such as for new trajectory guidance processes (performed by the specific *Trajectory Guidance* capability). Besides it updates data for planning or executing a specific trajectory (procedure attributes) and data about the state of these tasks (procedure states). 

**Figure 8:**
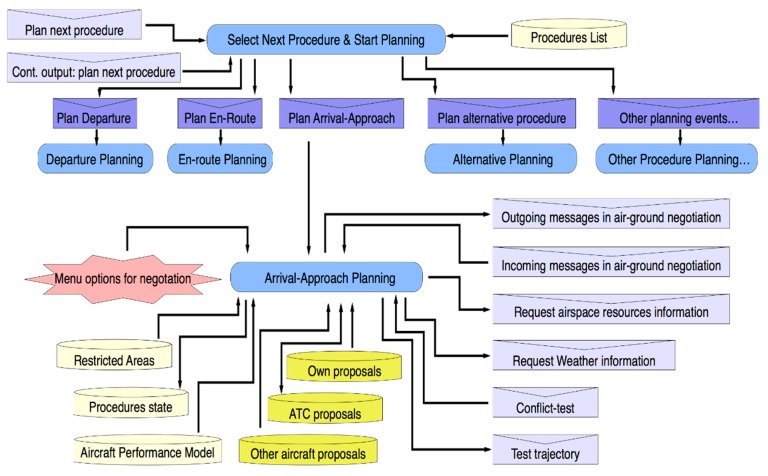
Next procedure planning capability overview.

The *Planning Next Procedure* capability is responsible for planning the trajectory linked to the next procedures on the procedures list. Figure 8 represents a simplified architecture of this sub-capability. It illustrates how plans that implement several processes of this sub-capability are organized. Moreover the Figure shows how events and data are used or generated by these plans. In this case, at first the plan named *Select Next Procedure and Start Planning* is triggered by *Plan Next Procedure* events, which come from either the *Procedure Event Execution* capability or the *Manage Contingence* capability. In turn, this plan initiates a new plan that executes planning tasks according to procedure data selected from the list. Thus, if the next procedure corresponds to the arrival and approach flight phase, then the *Arrival/Approach Planning* plan is initiated. Main inter-agent messages used and generated by this plan are shown in Figure 8, together with other messages (e.g., *Conflict-Test* or *Test Trajectory*) that are used at an interior level to exchange data with other capabilities. 

The *Re-Planning Current Procedure* capability contains a set of plans to modify the current trajectory attributes in several ways after a contingency is detected and informed by the *Airborne Contingency* capability through a contingency event. When a contingency event is received from this last capability, a plan for starting a new negotiation process is selected according to the information provided by the abovementioned event. 

### 3.2. Trajectory Guidance

**Figure 9:**
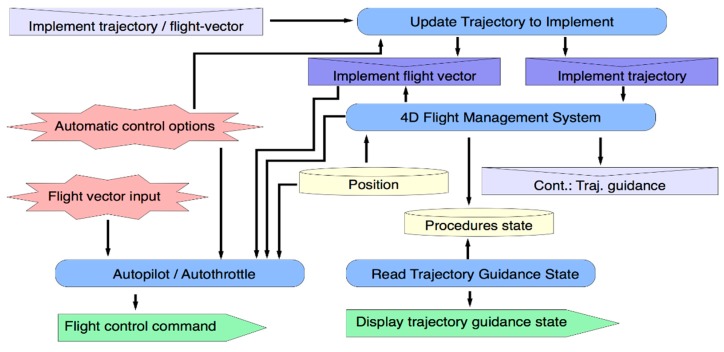
Trajectory Guidance Capability.

This capability (see Figure 9) represents a flight control system for trajectory guidance at several automation levels (3D/3.5D/4D) or for flight-vector guidance (Autopilot and/or Flight Director). Therefore, obtaining the full benefits of SATS requires extending functionalities of the current *Flight Management Systems* (*FMS)* and *Flight Director* [61] to perform 4D-trajectory guidance. It also provides information about the real-time guidance process, required by other capabilities or agents. Flight information about the trajectory to be flown by the aircraft is provided by the *Implement Trajectory/Flight-Vector* event that comes from the *Navigation Procedures Management* capability*.* Other inputs come from the user interface (e.g., automatic control options which define automation levels to execute trajectories, flight vector input or flight control inputs, *etc.*)*.*

Information about the real-time guidance process (trajectory state) is stored and contingency events are generated to identify guidance difficulties. Finally, aircraft actions are controlled through Flight Control Commands.

### 3.3. Aircraft Environment Information Management

The main goal of this capability consists of maintaining updated environmental knowledge on-board. Information is obtained from percepts of the aircraft sensor systems and from incoming *agent* messages. Plans on this capability update and store *data* information from: sensor data, weather forecasts, restricted areas, air space recourses (e.g., available arrival routes and gateways), surrounding air traffic, *etc.* This capability generates specific contingency events (represented as inner messages in Figure 6) when significant environmental changes are detected. These events will be dealt with by plans included in the Aircraft Systems Alarms Management that is described below. Moreover each source providing sensed information about environment will be conveniently modeled considering the associated uncertainty, update rate, *etc.*

Alarm system outputs are managed by this capability to inform about contingency events when aircraft system (avionics, engine, *etc.*) failures arise on-board. 

### 3.4. Conflict Detection-Resolution

As its name suggests, it is responsible for detecting conflicts with other aircraft or obstacles (terrain, adverse weather areas, *etc.*). It also provides a set of ranked proposals for conflict resolutions. Proposals are also negotiated and/or implemented through other capabilities. This capability is also used to allow *what-if* analysis of conflict resolution and to suggest solutions while planning trajectory processes. 

### 3.5. Airborne Contingency Management

This capability deals with deciding procedural tasks according to contingency *input events* received from other capabilities or external agents. The following contingencies inputs have been identified:

- Contingency of critical environmental changes.
- System failure contingency, indicating failure details as well as the proposed procedure, maneuver or actions according to normal, abnormal or emergency procedures.
- Conflict contingency, including information about solutions proposed by the *conflict detection-resolution* capability.
- Contingency from other aircraft (*i.e.*, requirements from other aircraft asking to solve conflicts, to modify arrival sequence, *etc.*).
- Airline contingency, asking to modify intended flight plan.
- Contingency from ATC (e.g., changes regarding previous agreement).
- Contingency related to an unexpected emergency due to crew or passengers defined through an on-board options menu.

Recommendable procedural tasks are suggested by means of *contingency events* in order to be considered during the current procedure (e.g., *modify executing procedure* event) or the next one (e.g., *plan next procedure* event). The contingency solving process can be treated in a partially automated manner and therefore the design of this capability requires work in the future to develop suitable new decision-making schemes with user intervention.

## 4. From On-Board Capabilities to Cockpit HCI System Architecture

As a result of previous aircraft agent design, a cockpit HCI system architecture has been achieved. As explained, it takes into account emerging CNS/ATM technology functionalities (e.g., Automatic Dependence Surveillance) and integrates them into capabilities that also contain new functionalities for performing more automated navigation procedures for the next-generation of SATS. 

**Figure 10:**
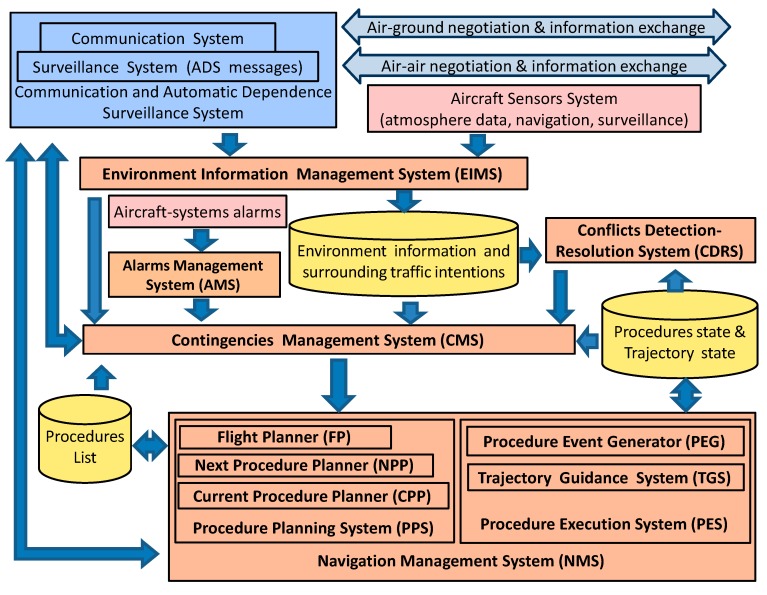
Cockpit HCI architecture.

This architecture is shown in Figure 10. According to above analysis, the sub-systems that constitute this architecture share and use the next three main data groups:

(a) *Environment and Surrounding Traffic Information.*
(b) *Procedures List.*
(c) *Procedures State.*

*Environment* and *Surrounding Traffic Information* data comes from the aircraft sensors system as well as the recent Communication and Automatic Dependence Surveillance prototypes (ADS) ([1]).

*Procedures List* data contains the sequence of procedures that aircraft should flight along its gate-to-gate route. For example, if the aircraft is planning the arrival process while a cruise procedure is been executed, both procedures will be put in consecutive position on the list.

Finally, *Procedures State* data capture/collects information about the management process for each procedure to indicate phases of planning, executing or modification of its associated trajectory. Therefore, they represent discrete states primarily used on board for the generation of events and for decision-making processes. In addition, this data can also be sent through aircraft state messages to others SATS agents, which in turn can use them on their inner processes (*i.e.*, the procedure state of an aircraft provides more complete information for ATC and surrounding aircraft monitoring and surveillance tasks). 

Previous data are shared by the next sub-systems that are part of the cockpit HCI. This architecture mainly matches aircraft agent capabilities. These sub-systems are the following:

(a) *Navigation Management System.*
(b) *Contingency Management System.*
(c) *Environmental Information Management System.*
(d) *Alarm Management System.*
(e) *Conflict Detection Resolution System.*
(f) *Communication and ADS System.*

The *Navigation Management System* (*NMS)* is responsible for planning and executing trajectories. It is modeled through the *Navigation Procedures Management* and the *Trajectory Guidance* capabilities. Then the NMS consists of two new sub-systems: the *Planning Procedure Systems* (PPS) and the *Executing* *Procedure System* (EPS). The first one is modeled by the *Flight Planning* capability, the *Planning Next Procedure* capability and the *Current Re-Planning* *Procedure* capability. The second one (EPS) is based on the *Event Procedure Generator* (which corresponds with the *Procedure Execution* Capability) and the *Trajectory Guidance System*, which is modeled by means of the *Trajectory Guidance* capability. 

Therefore, NMS represents the core of the proposed on-board architecture. This system extends functionalities of the current Flight Management Systems (FMS) and autopilot/autothrottle (AP/AT) to introduce a higher cockpit automation level for SATS in the following manner:

- The *Procedure Planning System* supports full procedure planning processes (that includes air-ground and air-air negotiation, requesting environment information, *etc.*) *versus* functionalities of current FMS for computing route parameters.
- *The Procedure Execution System* extends (through Trajectory Guidance System) the current flight plan guidance functionality of AP/AT to provide new functionalities for 4D-trajectory guidance, as is shown in Figure 9. The *Procedure-Event Generator* also represents a new level of information about an on-board HCI state for managing executing procedure and initiating new planning processes.

The *Contingence Management System* (CMS) maps the *Airborne* *Contingence Management* capability and therefore uses their associated events (*i.e.*, incoming messages, percepts from the pilot interface as well as contingence events from the rest of the systems). From these events and updated data, the CMS generates output contingence events aimed at implementing new solutions by the NMS. Events will also be provided by sensors and other sources conveniently modeled. Consequently, CMS represents the first level of a decision-making system above of the *Navigation Management* *System*. 

The *Environmental Information Management System* (EIMS) is responsible for updating environmental aircraft information as well as providing information about significant changes on it. This information is used by the *Conflict Detection and Resolution System (CDRS)* to generate specific contingence events to be treated by *Contingence Management System.* In the same way, *Alarms Management System* (AMS) generates contingence events for CMS when aircraft system alarms arise. EIMS, CDRS and AMS can be modeled through their respective capabilities (*i.e.*, *Aircraft Environment Information Management*, *Conflict Detection-Resolution* and *Aircraft Systems Alarm Management*).

## 5. Conclusions

In this paper we have presented an activity analysis aimed at designing HCI in avionic systems for a next-generation of Smart Air Traffic Systems. An agent-based methodological approach has proved its suitability for the integration of on-board activities, the HCI and underlying calculations in a conceptual model. The conceptual model describes the dynamic behavior and architecture of the entire SATS and their agents. The applied methodology has enabled a formal, structured and consistent descriptive analysis of HCI in SATS, particularly in on-board navigation tasks. Thus, by means of an iterative top to bottom modeling process, goals, and tasks from an aircraft management scenario have been organized on capabilities, internal events, plans, and data structure. This architecture is directed at the execution of several processes in order to plan, perform or modify trajectories in a coordinated way. 

Then, this work illustrates: (i) the core of a new cockpit system for managing navigation procedures; (ii) how agent (or cockpit) capabilities can integrate new capabilities, events, plans, data, *etc.*; and (iii) how future underlying mathematical models for a cockpit system can be implemented within the capability.

The modular design of the aircraft agent internal architecture around the concept of capability provides a direct union/connection between them and on-board systems for managing their respective procedures. *Procedure Management* capability together with *Trajectory Guidance and Contingency* capability build the core around which a future SATS Navigation Management Systems (NMS) could be developed. Thus NMS is described as an on-board system that includes flight planning and navigation guidance capabilities in current FMS, adding other features such as: (i) obtaining user preferred trajectories; (ii) performing automated trajectory negotiation processes; (iii) evaluating 4D trajectory proposals from other agents; (iv) generating new proposals for other agents; and (v) providing flight guidance along negotiated 4D trajectories.

Several aspects and directions for future works should be consider from the described conceptual model. We focus on the implementation issue, due to its importance into the life-cycle of the working model and validation process.

The present model can be useful as base for performing a distributed simulation by means of discrete events where the agent’s messages interchange defines events and integration mechanisms between systems. Continuous simulation is also possible when the aircraft dynamic is implemented for HITL and fast-time analytical simulation purposes.

The descriptors of final artifacts of our conceptual model provide enough details to translate it into a working model by means of current agent development tools and platforms. For example, Prometheus Development Tool (PDT) provides a full life-cycle support for: (i) designing most of the model components; (ii) automatic cross-checking for consistency and completeness of the conceptual model; and (iii) automatic generation of skeleton code in JACK agent-oriented programming language [60]. In addition to JACK, other agent platforms that provide FIPA standards infrastructure for inter-agent communications [64] (such as JADE [58] or JADEX [59]) are also suitable solutions for implementation issues. 
